# HIV Prevention, Care, and Treatment in Two Prisons in Thailand

**DOI:** 10.1371/journal.pmed.0040204

**Published:** 2007-06-26

**Authors:** David Wilson, Nathan Ford, Verapun Ngammee, Arlene Chua, Moe Kyaw Kyaw

## Abstract

The authors discuss the challenges of providing HIV treatment to a marginalized population: prisoners in Thailand.

As scale-up of antiretroviral therapy (ART) progresses in less-developed countries, the challenges of providing treatment to marginalised populations become of increasing concern. One such marginalised group is prisoners. While there is an emerging consensus that prevention and treatment is feasible and effective in prisons [1], experience of implementing comprehensive HIV/AIDS programmes that include antiretroviral therapy in resource-limited countries is limited. This article describes our experience of providing HIV prevention and treatment in two prisons in Thailand.

## HIV in Prisons in Thailand

Thailand is noted for its successful response to the HIV epidemic. Successful prevention efforts achieved an 83% reduction in new infections between 1990 and 2003, and the country can be said to have achieved the goal of universal access to antiretroviral therapy, with about 76,000 people on treatment out of a total of roughly 600,000 people with HIV [2]. However, several groups have been unable to access the government's treatment programme, in particular injecting drug users, migrants, and prisoners (the first two groups contributing significantly to the third) [3].

A reported 168,264 people are incarcerated in Thailand (point prevalence), exceeding prison capacity by over 50% [4]. Prison data on HIV prevalence are sparse—testing is not performed systematically and no random surveys have been done—but what data do exist indicate that prevalence is much higher than in the general population [5]. One study in Klong Prem Central Prison, Bangkok, found that 25% of prisoners who agreed to be tested (*n* = 689; convenience sample) were HIV positive [6], compared to a general prevalence of 1.5% in the national population [2].

Thailand's prisons suffer from lack of health staff and severe budget constraints. The health care workforce in the 139 prisons across Thailand comprises just 17 full-time and 16 part-time doctors and 307 nurses. In 2000, Thailand spent over US$150 million on prison health care (including infrastructure-related costs), but less than US$25,000 was spent on HIV/AIDS care [7]. Until October 2005, the total annual health budget per prisoner was around US$3.5, disbursed through the Department of Corrections. Since then, the prison health budget has come under the administration of the National Health Security Office's health insurance scheme. This scheme covers treatment for most health conditions, including HIV/AIDS, thus removing budgetary constraints for the treatment of most patients. However, health insurance is only available to registered Thai nationals. Around 5% of the total prison population are foreign nationals, mostly from Myanmar, and an additional unknown but significant number are unregistered Thais (ethnic minorities, nationals without birth registration, or those who have lost their identity cards) [8]. In addition, the health insurance scheme does not fund prevention, education, or other non-curative activities.

## HIV/AIDS Care in Two Prisons in Bangkok

Médecins Sans Frontières (MSF) has supported HIV/AIDS programmes in Thailand since 1995. In June 2003, at the invitation of the prison health services, we began providing clinical support in two prisons in Bangkok—Minburi, a remand prison, and Bangkwang, a maximum-security prison (Box 1). The initial focus was on treatment, but once a level of trust was built between MSF and prison health authorities, we were invited to expand our work to prevention activities. The following observations are derived from these programmes.

**Box 1. Minburi and Bangkwang Prisons**

Minburi Remand Prison is a medium-security prison with a capacity of 2,000 (1,700 males and 300 females). The duration of sentence is two to seven years. Health care staff are one part-time doctor and three full-time nurses. Prisoners needing hospitalisation are transferred to Klang Hospital, a 300-bed hospital in Klong Prem Central Prison, Bangkok. MSF began working in Minburi Remand Prison in June 2003.

Bangkwang is a maximum-security prison. Its official capacity is 3,500 (males only), but in March 2006 it held 4,922 prisoners, including 870 on death row. The minimum sentence is 25 years. There is a 40-bed hospital and an outpatient clinic. Health services are staffed by one full-time doctor (director of prison medical services) and another part-time doctor, six registered nurses, six technical nurses, two pharmacists, one laboratory technician, and two x-ray technicians. MSF began working in Bangkwang in December 2004.

Both prisons are overcrowded. Prisoners occupy large communal rooms. In Minburi there are about 400 prisoners per room in the male prison and 300 per room in the female prison. In Bangkwang there are 20 to 40 prisoners per room, except on death row, where there are about 100 per room. A small number of prisoners are held in solitary confinement.

In both prisons, all inmates are locked in their cells from 4 pm to 7 am, during which time health staff do not access the cells. In Bangkwang, “first aid” health care is provided by designated prisoner volunteers during these hours.

## Prevention

Drug users in Thailand are the highest risk group for HIV infection. Around one-fifth of all new HIV infections occur through injecting drug use; in some parts of the country this figure rises to above 50% [3]. Up to two-thirds of prisoners are incarcerated for drug-related offences [2]; some, but not all, are injecting drug users, and this contributes to the high prison HIV prevalence. Injecting drug use in the prisons has decreased in recent years, partly because of reduced availability of heroin (inside and outside). Where it does occur, injecting equipment is scarce and almost always shared. Some prisoners tell us that they used to belong to an “injecting group” but this stopped when other drug users started to die from AIDS.

Tattooing is another risk factor for HIV infection. In Thai prisons, tattooing equipment is prohibited and therefore it is often shared. Sharpened pens or sewing needles may be used for tattooing and there are no means for sterilisation.

But by far the greatest risk factor for HIV transmission within Thai prisons is unprotected sex between men. Sex, consensual or otherwise, is part of prison life (Box 2). Condoms are not banned from prisons, but the attitude of prison staff towards sex between prisoners influences condom distribution.

**Box 2. Sex in Prisons**

**What kinds of sex occur in all-male prisons?**

- Sex in the prisons is frequently related to power.
- *Consensual sex* between men includes partner sex, paid sex, and sex in exchange for protection.
- *Non-consensual* sex includes rape, sometimes used as an intimidation tactic, and coerced sex as repayment of debt. New prisoners are particularly vulnerable as they can be subjected to sex as an initiation to the power relations in the prison.

**Why may the attitude of prison staff prevent safer sex practices?**

- Some prison staff try to forbid all sexual activities in order to prevent non-consensual sex; others believe that forbidding sex is the most effective form of prevention, though in practice this is unrealistic.

**Why do prisoners fail to use condoms?**

- Condoms are not banned in prison, but in practice it is difficult to gain acceptance from prison guards for condom distribution.
- Some prisoners are unaware of the risk of HIV transmission by anal sex. Others are aware, but are unable to access condoms or to negotiate their use in coercive situations.
- Being known to have sex or to possess condoms may lead to criticism by prison guards or ostracism by other prisoners. Prisoners may prefer to risk becoming HIV positive than to try to access condoms.
- Prisoners may believe that having sex with another man is against nature. This leads them to be secretive and have clandestine, unprotected sex.
- Someone who insists on condom use can give the impression that they are HIV positive or have a sexually transmitted infection.

The governors of both prisons where we work gave approval for condom distribution during 2006, but distributing them widely depends on changing attitudes of prison staff. While health staff are generally supportive, the engagement of other staff is essential to move distribution beyond the clinics. Prison guards want to see HIV transmission reduced, but often find it hard to accept that for this to occur they must play an active role in condom distribution. Progress has been made in both prisons through workshops in which prison staff are encouraged to question their own attitudes and behaviour towards prisoners, through activities such as role-plays. In Bangkwang condoms are available through prisoner representatives (prisoners designated by the Director of Medical Services and trained by MSF). In Minburi, where there is a rapid turnover of prisoners, prisoner representatives are less easy to establish and the focus is on prison guards, many of whom now agree to distribute condoms.

Workshops for prisoners give information about HIV transmission and prevention. These workshops also provide an opportunity for participants to share their experiences and learn from each other about how to solve the difficulties of establishing safer sexual practices in the prison. Such a participatory approach is also part of the development of mutual support amongst the prisoners.

## HIV Testing and Counselling

In the past, prisoners generally received no pre- or post-test counselling. Health care staff may have avoided informing prisoners of their status because treatment was unavailable. We have seen several patients whose medical record showed that an HIV diagnosis had been made several years ago but the patients had not been informed of their status. Counselling is now implemented in both prisons, but not all prison staff believe it is necessary and more work is needed to explain its benefits. Confidentiality is a major challenge and we have attempted to address this issue during workshops for both staff and prisoners. Nevertheless, during the first 18 months of the programme, 20 prisoners failed to attend any follow-up counselling after receiving a positive HIV diagnosis.

Most patients we have seen to date (112 from a total of 165) were diagnosed with HIV infection while in prison, with diagnosis most often prompted by an opportunistic infection (81 patients) such as pulmonary tuberculosis (45 patients). Some former injecting drug users sought testing when other drug users died.

## Care and Treatment for HIV/AIDS

Clinical support began with the treatment of opportunistic infections. During this phase—lasting six months in both prisons—prison medical staff and MSF jointly set up a peer support system for adherence to antiretroviral treatment.

Of 165 HIV-infected prisoners identified since the program began, 122 (74%) were in disease stage 3 or 4 as defined by the World Health Organization (WHO). Most opportunistic infections have been manageable within the prison, including pulmonary tuberculosis (43 cases) and extra-pulmonary tuberculosis (28 cases). Two patients with multidrug-resistant tuberculosis, two with cryptococcal meningitis, and two with cytomegalovirus retinitis have been referred to an infectious diseases hospital for diagnosis and treatment. Two patients with pneumonia have died after referral and hospital admission. There is no access to treatment for hepatitis B and C in the prisons and we do not routinely test for these conditions. Even outside the prison availability of such treatment is extremely limited.

Medical criteria to initiate antiretroviral therapy follow Thai national guidelines: CD4 count below 250 cells/mm^3^ for patients with WHO stage 2, 3, or 4 disease, or below 200 in asymptomatic cases. Patients are provided with basic knowledge about HIV, opportunistic infections, ART, and the importance of adherence so that they can make informed decisions regarding whether and when to start treatment. If patients proceed to treatment, antiretroviral therapy is administered by a nurse helper—a volunteer prisoner who helps medical staff with tasks such as dispensing medications and checking temperature, weight, and blood pressure. The first-line regimen for 87 patients (75%) is a fixed-dose combination of stavudine, lamivudine, and nevirapine [9]. Patients with tuberculosis co-infection who are taking rifampicin are prescribed stavudine, lamivudine, and efavirenz.

We have enrolled 88 patients on antiretrovirals, of whom 12 (15%) had previously taken ART but discontinued it when they were imprisoned (Table 1). The median follow-up time on treatment is 18 months and CD4 cell gain is comparable with treatment programmes in other settings [9]. Currently we follow 63 patients on ART (72%) within the prisons. Of the remaining patients, 18 (20%) have been released from prison and five (6%) were transferred to other prisons; two patients (2%) have died.

**Table 1:**
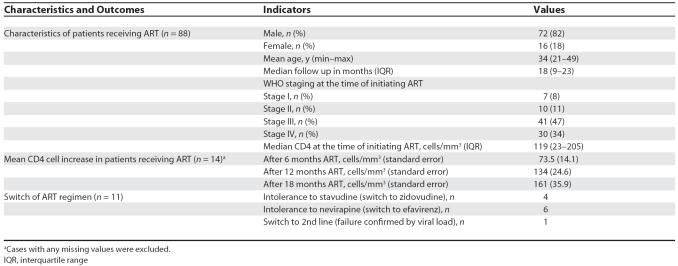
Characteristics and Outcomes of Patients with HIV Receiving ART in Two Prisons

Peer support from people living with HIV/AIDS is part of Thailand's treatment strategy [10], and peer support systems have been established in both prisons. In addition to this group support, patients are assigned a buddy to support adherence to medication (the buddy is normally someone who has HIV and who may also be on ART).

Prisons have been said to be the ideal environment for ensuring high compliance to treatment [11], but in our experience there are many important barriers to overcome that require constant re-evaluation. These include fear of stigma associated with HIV, which discourages prisoners from taking medicines in a crowded environment; mistrust of prison staff, including hospital staff; transfer to another facility; and poor social support upon release.

The attitude of the prison health care staff towards the rights of prisoners to access ART is very positive. Staff are committed to working through difficult problems and referring people to other hospitals in cases where specialist care is needed. The fact that none have questioned that three prisoners facing the death sentence receive antiretroviral treatment is a clear indication of the staff's commitment to treatment as a basic human right.

This positive attitude is crucial to success in a setting where the traditional relationship between prisoners and prison staff is antagonistic: in the past prisoners distrusted staff and were unwilling to come forward for care. According to the medical director in Bangkwang, it took two years to gain the trust of patients before they were willing to seek treatment.

## Patient Transfer and Release

Bangkok prisons are overcrowded and transfers to less crowded prisons elsewhere in the country are common. Such transfers create problems for ensuring continuity of treatment because most prisons outside Bangkok have limited health staff and no access to HIV treatment. While efforts are made to communicate the particular health needs of people with HIV/AIDS within the prison system, the reality is that we do not know what care is provided for the five patients from our cohort who have been transferred to other prisons.

Continuity of care upon release is also challenged by substance use, homelessness, joblessness, poverty, and difficulties in accessing the health insurance scheme, especially for ex-prisoners without an ID card (Box 3). Before being released from prison, patients meet with a social worker employed by MSF to develop a plan for continuing treatment. In both prisons, MSF provides a medication supply for three months—the average time taken for people with a correct Thai ID card to enrol in the government ART program. Of 18 prisoners released since the start of the treatment programme, seven receive treatment through the public health system, while six receive treatment from MSF because they lack a Thai ID card. Despite considerable efforts, five have been lost to follow up.

**Box 3. Ensuring Continuity of Care after Release from Prison: Case Studies**

The first patient is a 33-year-old Thai man who lost his ID card many years ago. He was found to be HIV positive whilst in Minburi Remand Prison and began antiretroviral therapy in May 2004. He contacted MSF many times for reassurance that he could continue treatment after his release.

He was released in October 2005, stayed with friends to whom he did not disclose his HIV status, and worked as a welder. MSF supplied his medication and arranged lab monitoring. Documentation he needed in order to apply for an ID card included his birth certificate, his father's death certificate, a certificate of release from prison, proof of residence—which required the house owner to add his name to a household registration document—and two certificates of good character signed by state employees. MSF helped him through the necessary bureaucratic procedures and he received his ID card in February 2007—16 months after release. This enabled him to register with the National Health Insurance scheme and, after a further three months, receive treatment through the government program.

The second patient is a 30-year-old man diagnosed with HIV in 2002 in Minburi Remand Prison. He did not attend the clinic while in prison but asked MSF to visit him upon his release in May 2004. Subsequently he was diagnosed with miliary tuberculosis and then commenced ART in May 2005 after MSF helped link him to the government ART program. He had previously injected drugs and started again in June 2005. However, his adherence to ART remains good.

## Ethical Considerations Surrounding This Article

Ethical questions inevitably arise during any intervention amongst incarcerated populations. In this paper we have described our experience of implementing proven HIV prevention strategies and delivering standard treatment to a difficult-to-reach population requiring an innovative approach. The main ethical challenges we have faced, together with our partners, have been the implementation of practices of confidentiality and informed consent; we have done our best to rise to these challenges.

During the peer review process for this article, a number of questions related to ethics were raised, specifically concerns about patient anonymity and the need for ethical approval for such a publication. The matter was referred to the PLoS Medicine Advisory Group on Publication Ethics, who were divided on the matter [12]. We referred the issue to an independent institutional review board, whose judgement was that there was minimal risk of harming individuals or the community by publishing this paper. The board also felt that as a descriptive paper in which no research hypothesis was being tested and no analytic statistics were used, there was no requirement for formal ethics committee review and approval.

## Discussion

There are few examples of HIV/AIDS treatment programmes in prisons in the developing world. Emerging outcomes from pilot programmes support the effectiveness of treatment in prisons [13–15], but poor or non-availability of antiretrovirals is still frequently reported [16–18]. This lack of availability has become a growing concern for treatment activists: in South Africa, civil society groups have recently taken the government to court to fight for prisoners' rights to treatment [19].

In our experience, satisfactory outcomes can be achieved in under-resourced prison settings. MSF's initial input was to provide treatment for opportunistic infections, antiretroviral drugs, and technical support. Thailand is not a “least-developed” country, but the approach taken follows WHO guidelines for treatment in resource-poor settings, and could be implemented by any government providing treatment in the general population. Ultimately, barriers that prevent the provision of treatment in prisons when it is available outside are not technical or financial, but political.

The effectiveness of any prison programme depends on the attitude of staff. We have found the attitude of prison health staff to be very positive towards the rights of prisoners, but this is not yet the case for all staff. Treatment provision was an important first step towards building sufficient trust that then allowed us to bring up the more sensitive issue of prevention.

We believe the programme will have a sustained impact. The prevention component includes strategies to change the attitudes and behaviour of both prison guards and prisoners, and the Department of Corrections has asked us to develop training curricula to be used in other prisons. In terms of treatment, drug supply has been handed over to the government and training and mentoring has provided prison medical staff with the skills to manage most problems. Sustainability also comes from the fact that part of the budget for health care for Thai prisoners, including antiretroviral medicines, now comes under the health insurance scheme. However, Thai ethnic minorities and foreigners are not eligible for this support and their access to care is a pressing concern.

Most prisoners come from marginalised groups such as drug users, sex workers, or unregistered migrants. These groups are at high risk of HIV with limited access to health care in general, and this is reflected in the high number of HIV-positive cases diagnosed in the prison. Implementing treatment and prevention programmes in prisons provides an opportunity to work with particular groups who would not normally seek, or be given, care. An effective response must therefore confront barriers to care outside of prisons, in order to ensure continuity of care for those who are released.

## Supporting Information

Alternative Language Text S1 Thai translation of the article by K. Kijitiwatchakul (201 KB DOC).

## Abbreviations

ART: antiretroviral therapy
MSF: Médecins Sans Frontières
WHO: World Health Organization
