# Supplementary material for: HIV Prevention, Care, and Treatment in Two Prisons in Thailand
Source: PLoS Med. 2007 Jun 26;4(6):204. doi: 10.1371/journal.pmed.0040204 (PMC1896202; doi:10.1371/journal.pmed.0040204)
Supplement: Alternative Language Text S1 — (201 KB DOC). [file pmed.0040204.sd001.doc]

HEALTH IN ACTION

**การป้องกัน ดูแลและรักษาผู้ติดเชื้อเอชไอวีในเรือนจำสองแห่งในประเทศไทย**

**เดวิด วิลสัน, นาธาน ฟอร์ด, วีระพันธ์ งามมี, อาร์ลีน ชัว, โม โจ โจ้**

**การสนับสนุนงบประมาณ**

โครงการนี้ได้รับการสนับสนุนงบประมาณจากการบริจาคของบุคคลต่างๆ ที่ให้กับ MSF ส่วนยารักษาได้รับการสนับสนุนจากกระทรวงสาธารณสุขและกองทุนของ MSF โดยใช้ยาสามัญที่ผลิตได้ในประเทศเท่าที่ทำได้

# ผลประโยชน์ทับซ้อน ผู้เขียนยืนยันว่าไม่มีผลประโยชน์ทับซ้อนใดๆ

**อ้างอิง** Wilson D, Ford N, Ngammee V, Chua A, Kyaw MK (2007) HIV prevention, care, and treatment in two prisons in Thailand. PLoS Med 4(6): e204. doi:10.1371/journal.pmed.0040204

**ลิขสิทธิ์**: © 2007 วิลสันและคณะ บทความชิ้นนี้เผยแพร่ต่อสาธารณะภายใต้เงื่อนไขของ Creative Commons Attribution License ซึ่งอนุญาตให้ใช้ แจกจ่าย และผลิตซ้ำได้ในสื่อทุกรูปแบบ โดยต้องระบุชื่อผู้เขียนและที่มา

**คำย่อ** ART ยาต้านไวรัส, MSF องค์การหมอไร้พรมแดน, WHO องค์การอนามัยโลก

ผู้เขียนทั้งหมดเป็นเจ้าหน้าที่*องค์การหมอไร้พรมแดน* (Médecins Sans Frontières - MSF) *บางกะปิ กรุงเทพฯ*

*อีเมล์* : [msfb-bangkok@brussels.msf.org](mailto:msfb-bangkok@brussels.msf.org)

ขณะที่การเพิ่มระดับการรักษาด้วยยาต้านไวรัส (antiretroviral therapy - ART) ในประเทศกำลังพัฒนากำลังคืบหน้าไปด้วยดี แต่การให้การรักษาแก่ประชากรชายขอบกลุ่มต่างๆ กลับเป็นประเด็นที่น่ากังวลมากขึ้นเรื่อยๆ หนึ่งในกลุ่มดังกล่าวคือ กลุ่มผู้ต้องขัง แม้จะมีความเห็นพ้องต้องกันอยู่ว่าการป้องกันและการรักษาในเรือนจำนั้นสามารถทำได้และมีประสิทธิผล [1] แต่ประสบการณ์ในการดำเนินโครงการเอชไอวี/เอดส์ที่มีการรักษาด้วยยาต้านไวรัสในประเทศที่มีทรัพยากรจำกัดนั้นมีอยู่ไม่มากนัก บทความนี้จะอธิบายถึงประสบการณ์ของเราในการดำเนินการป้องกันและรักษาผู้ติดเชื้อเอชไอวีในเรือนจำสองแห่งในประเทศไทย

**สถานการณ์เอชไอวีในเรือนจำในประเทศไทย**

ประเทศไทยได้ชื่อว่าประสบความสำเร็จในการรับมือกับการแพร่ระบาดของเชื้อเอชไอวี มาตรการป้องกันต่างๆ ประสบความสำเร็จในการลดการติดเชื้อใหม่ได้ถึง 83% ในช่วงปี 2533 ถึง 2546 และกล่าวได้ว่าไทยสามารถบรรลุเป้าหมายของการเข้าถึงการรักษาด้วยยาต้านไวรัสแก่ผู้ติดเชื้อประมาณ 76,000 คนจากทั้งหมดราว 600,000 คน [2] แต่อย่างไรก็ตาม ยังมีอีกหลายกลุ่มที่ไม่สามารถเข้าถึงโครงการรักษาของรัฐได้ โดยเฉพาะกลุ่มผู้ติดยาเสพติด แรงงานข้ามชาติ และผู้ต้องขัง (สองกลุ่มแรกคือคนส่วนใหญ่ในกลุ่มที่สาม) [3]

ผู้ต้องขังในประเทศไทยตามรายงานมีจำนวนถึง 168,264 คน ซึ่งเกินความสามารถในการรองรับของเรือนจำถึง 50% [4] ข้อมูลเรื่องการติดเชื้อเอชไอวีของเรือนจำนั้นก็มีน้อยมาก ไม่มีการตรวจอย่างเป็นระบบ และไม่เคยมีการสำรวจสุ่มตัวอย่าง แต่ข้อมูลเท่าที่มีอยู่ก็บ่งชี้ว่าสัดส่วนจำนวนผู้ติดเชื้อในหมู่ผู้ต้องขังนั้นมีสูงกว่าประชากรทั่วไปมาก [5] การศึกษาชิ้นหนึ่งที่ทำในเรือนจำคลองเปรม กรุงเทพฯ พบว่า 25% ของผู้ต้องขังที่สมัครใจรับการตรวจ (689 คน, สุ่มตัวอย่างตามความสะดวก) ติดเชื้อเอชไอวี [6] ในขณะที่สัดส่วนการติดเชื้อของประชากรทั่วไปคือ 1.5%[2]

เรือนจำในประเทศไทยประสบปัญหาขาดแคลนบุคลากรด้านการแพทย์และงบประมาณที่จำกัด บุคลากรด้านการแพทย์ในเรือนจำ 139 แห่งทั่วประเทศมีแพทย์ทำงานเต็มเวลาเพียง 17 คน แพทย์ทำงานไม่เต็มเวลา 16 คนและพยาบาล 307 คน ในปี 2543 ประเทศไทยใช้งบประมาณกว่า 150 ล้านเหรียญสหรัฐฯในการดูแลสุขภาพผู้ต้องขัง (รวมค่าใช้จ่ายที่เกี่ยวกับโครงสร้างพื้นฐานด้วย) แต่ใช้ไปกับการรักษาเอดส์ไม่ถึง 25,000 เหรียญสหรัฐฯ [7] งบประมาณด้านสุขภาพที่คำนวณจนถึงเดือนตุลาคม 2548 ที่เบิกจ่ายผ่านกรมราชทัณฑ์ก็คือ ราว 3.5 เหรียญสหรัฐฯ ต่อผู้ต้องขังหนึ่งรายต่อปี หลังจากนั้น งบประมาณด้านสุขภาพสำหรับผู้ต้องขังถูกบริหารจัดการโดยโครงการประกันสุขภาพของสำนักงานหลักประกันสุขภาพแห่งชาติ โครงการนี้ครอบคลุมการรักษาพยาบาลเกือบทั้งหมด รวมถึงเอชไอวี/เอดส์ จึงเป็นการแบ่งเบาภาระทางงบประมาณในการรักษาผู้ป่วยส่วนใหญ่ อย่างไรก็ตาม การประกันสุขภาพครอบคลุมแต่เพียงผู้ที่มีสัญชาติไทยเท่านั้น ประมาณ 5% ของผู้ต้องขังทั้งหมดเป็นชาวต่างชาติ ส่วนใหญ่เป็นชาวพม่า และอีกส่วนหนึ่งที่ไม่ทราบจำนวนแน่ชัดเป็นคนไทยไร้สัญชาติจำนวนมาก (ชนกลุ่มน้อย คนที่ไม่มีสูติบัตร หรือคนที่ทำบัตรประจำตัวประชาชนหาย) [8] นอกจากนี้ โครงการประกันสุขภาพไม่ได้ให้การสนับสนุนการป้องกัน การให้การศึกษา และกิจกรรมอื่นๆ ที่ไม่ใช่การรักษา

**การรักษาผู้ติดเชื้อเอชไอวี/เอดส์ในเรือนจำสองแห่งในกรุงเทพฯ**

องค์การหมอไร้พรมแดน (Médecins Sans Frontières-MSF) ได้ให้การสนับสนุนโครงการเอชไอวี/เอดส์ในประเทศไทยมาตั้งแต่ปีพ.ศ. 2538 ในเดือนมิถุนายน 2546 ด้วยคำเชิญจากหน่วยงานด้านสุขภาพของกรมราชทัณฑ์ เราเริ่มให้การสนับสนุนทางการแพทย์แก่เรือนจำสองแห่งในกรุงเทพฯ คือ เรือนจำพิเศษมีนบุรีและเรือนจำกลางบางขวาง (ล้อมกรอบ 1) โดยเริ่มแรกมีจุดเน้นอยู่ที่การรักษาพยาบาล แต่เมื่อเกิดความไว้เนื้อเชื่อใจกันระหว่าง MSF และเจ้าหน้าที่ทางการแพทย์ของเรือนจำ จึงได้ขยายไปถึงกิจกรรมการป้องกันด้วย ข้อมูลดังต่อไปนี้ได้มาจากโครงการดังกล่าว

**การป้องกัน**

ผู้ใช้ยาเสพติดเป็นกลุ่มที่เสี่ยงต่อการติดเชื้อเอชไอวีมากที่สุดในประเทศไทย ประมาณหนึ่งในห้าของจำนวนผู้ติดเชื้อรายใหม่เกิดจากการใช้เข็มฉีดยาเสพติด ในบางพื้นที่สัดส่วนอาจเพิ่มสูงกว่า 50%[3] ผู้ต้องขังราวสองในสามเป็นผู้กระทำผิดในคดีที่เกี่ยวข้องกับยาเสพติด[2] โดยบางคนเป็นผู้ติดยาเสพติดแบบใช้เข็ม ทำให้อัตราการติดเชื้อในเรือนจำอยู่ในระดับที่สูง การฉีดยาเสพติดในเรือนจำลดลงในช่วงหลายปีที่ผ่านมา ส่วนหนึ่งเป็นเพราะเฮโรอีนหาได้ยากขึ้น (ทั้งในเรือนจำและภายนอก) แต่ในที่ที่มีการฉีดยาเสพติด อุปกรณ์หายากและมักจะใช้ร่วมกัน ผู้ต้องขังบางรายบอกเราว่าพวกเขาเคยอยู่ใน “กลุ่มฉีด” แต่ก็เลิกรากันไปเมื่อผู้เสพรายอื่นๆ เริ่มทยอยเสียชีวิตจากโรคเอดส์

การสักร่างกายก็เป็นปัจจัยเสี่ยงต่อการติดเชื้ออีกประการหนึ่ง อุปกรณ์การสักเป็นสิ่งต้องห้ามในเรือนจำของไทย ผู้ต้องขังจึงมักต้องใช้ร่วมกัน บางทีก็ใช้ปากกาที่ฝนปลายให้แหลมหรือเข็มเย็บผ้า และไม่มีวิธีการฆ่าเชื้อ

แต่ปัจจัยเสี่ยงสูงสุดในการแพร่เชื้อเอชไอวีในเรือนจำไทยคือการมีเพศสัมพันธ์ที่ไม่มีการป้องกันระหว่างผู้ต้องขัง เพศสัมพันธ์ ไม่ว่าจะด้วยความยินยอมหรือไม่ก็ตาม เป็นส่วนหนึ่งของชีวิตในเรือนจำ (ล้อมกรอบ 2) ถุงยางอนามัยไม่ใช่สิ่งของต้องห้ามในเรือนจำ แต่ทัศนคติของผู้คุมที่มีต่อการมีเพศสัมพันธ์ระหว่างผู้ต้องขังทำให้การแจกจ่ายถุงยางเอาแน่เอานอนไม่ได้

ผู้บัญชาการเรือนจำทั้งสองแห่งที่เราทำงานอยู่อนุมัติให้มีการแจกจ่ายถุงยางอนามัยในช่วงปี 2549 แต่การจะแจกจ่ายให้ทั่วถึงนั้นขึ้นอยู่กับการเปลี่ยนทัศนคติของเจ้าหน้าที่เรือนจำ และขณะที่เจ้าหน้าที่ทางการแพทย์นั้นโดยทั่วไปให้การสนับสนุน แต่ก็ยังต้องอาศัยความร่วมมือจากเจ้าหน้าที่ส่วนอื่นๆ ในการแจกจ่ายที่กว้างไปกว่าเฉพาะในคลีนิก บรรดาผู้คุมต่างต้องการให้การแพร่เชื้อเอชไอวีลดน้อยลง แต่ก็ยากที่จะทำใจยอมรับที่ตนเองต้องมีบทบาทแข็งขันในการแจกจ่ายถุงยาง มีการจัดการอบรมเชิงปฏิบัติการหลายครั้งในเรือนจำทั้งสองแห่ง โดยใช้วิธีการอย่างเช่นบทบาทสมมติ (role-play) ที่ให้เจ้าหน้าที่เรือนจำตั้งคำถามต่อทัศนคติและพฤติกรรมของตนเองที่มีต่อผู้ต้องขัง ที่บางขวางมีการแจกจ่ายถุงยางโดยตัวแทนผู้ต้องขัง (ที่ได้รับมอบหมายจากผู้อำนวยการด้านการแพทย์และผ่านการอบรมจาก MSF) ส่วนที่มีนบุรี ผู้ต้องขังต้องเปลี่ยนหน้าไปอยู่เรื่อยๆ จึงหาตัวแทนได้ไม่ง่าย ดังนั้นจึงมุ่งไปที่ผู้คุม ซึ่งตอนนี้ก็มีหลายคนที่ตกลงจะทำหน้าที่แจกจ่ายถุงยาง

การจัดการอบรมเชิงปฏิบัติการให้ผู้ต้องขังทำให้ได้ข้อมูลเกี่ยวกับการแพร่เชื้อเอชไอวีและการป้องกัน และยังได้เปิดโอกาสให้ผู้เข้าร่วมได้แบ่งปันประสบการณ์และเรียนรู้จากกันและกันถึงการมีเพศสัมพันธ์ที่ปลอดภัยมากขึ้นในเรือนจำ แนวทางการมีส่วนร่วมนี้ยังเป็นส่วนหนึ่งของการพัฒนาการช่วยเหลือซึ่งกันและกันในหมู่ผู้ต้องขังอีกด้วย

**การตรวจเชื้อเอชไอวีและการให้คำปรึกษา**

เมื่อก่อนนี้ ผู้ต้องขังมักไม่ได้รับการให้คำปรึกษาทั้งก่อนหรือหลังการตรวจ เจ้าหน้าที่ทางการแพทย์อาจจะหลีกเลี่ยงที่จะบอกผู้ต้องขังให้ทราบถึงสถานะของตนเองเนื่องจากไม่มีการรักษาพยาบาลให้ และเราได้เห็นผู้ป่วยหลายรายที่เวชระเบียนระบุว่ามีการตรวจพบเชื้อเอชไอวีเมื่อหลายปีก่อน แต่ผู้ป่วยไม่ได้รับการแจ้งให้ทราบ ตอนนี้มีการให้คำปรึกษาในเรือนจำทั้งสองแห่ง แต่ไม่ใช่เจ้าหน้าที่เรือนจำทุกคนจะเชื่อว่ามันเป็นสิ่งจำเป็น จึงจะต้องอาศัยความพยายามอีกมากที่จะอธิบายถึงข้อดีให้พวกเขาทราบ การเก็บข้อมูลเป็นความลับก็เป็นประเด็นสำคัญ และเราได้พยายามกล่าวถึงเรื่องนี้แล้วในการอบรมเชิงปฏิบัติการสำหรับเจ้าหน้าที่และผู้ต้องขัง ถึงกระนั้น ในช่วง 18 เดือนแรกของโครงการ มีผู้ต้องขัง 20 คนที่ไม่มาเข้าร่วมการให้คำปรึกษาหลังจากพบว่าตนเองติดเชื้อเอชไอวี

ผู้ป่วยส่วนใหญ่ที่เราพบจนถึงขณะนี้ (112 คน จากทั้งหมด 165 คน) พบว่าติดเชื้อเอชไอวีขณะต้องขัง โดยส่วนใหญ่มีการตรวจเนื่องจากป่วยเป็นโรคฉวยโอกาส (81 ราย) เช่น วัณโรค (45 ราย) ผู้เคยติดยาเสพติดบางรายก็มาตรวจหลังจากรู้ว่าผู้เสพยารายอื่นเสียชีวิตไป

**การดูแลรักษาผู้ติดเชื้อเอชไอวี/เอดส์**

การรักษาทางการแพทย์เริ่มจากการรักษาโรคฉวยโอกาส ในระยะนี้ซึ่งกินเวลาหกเดือนในเรือนจำทั้งสองแห่ง เจ้าหน้าที่การแพทย์ของเรือนจำและ MSF ร่วมกันจัดตั้งระบบช่วยเหลือกันเองในหมู่ผู้ติดเชื้อในการรักษาด้วยยาต้านไวรัส

จากผู้ต้องขังที่ติดเชื้อจำนวน 165 รายที่พบนับแต่เริ่มโครงการ มี 122 ราย (74%) ที่จัดอยู่ในระยะที่ 3 หรือ 4 ตามการกำหนดขององค์การอนามัยโลก โรคฉวยโอกาสส่วนใหญ่นั้นสามารถรักษาได้ภายในเรือนจำ เช่น วัณโรคในปอด (43 ราย) และวัณโรคนอกปอด (28 ราย) ผู้ป่วยวัณโรคดื้อยาหลายตัว 2 ราย เชื้อราในเยื่อหุ้มสมอง 2 ราย และ ติดเชื้อ CMV ที่จอประสาทตา อีก 2 รายถูกนำส่งโรงพยาบาลเพื่อทำการวินิจฉัยและรักษา ผู้ติดเชื้อที่เป็นโรคปอดบวม 2 รายเสียชีวิตหลังถูกนำส่งโรงพยาบาล ภายในเรือนจำไม่มีการรักษาโรคตับอักเสบบีและซี และเราก็ไม่ได้ทำการตรวจโรคนี้เป็นประจำ กระทั่งภายนอกเรือนจำการรักษาโรคนี้ก็มีอยู่จำกัดมาก

เกณฑ์การพิจารณาเริ่มการรักษาด้วยยาต้านไวรัสนั้นถือปฏิบัติตามแนวทางระดับประเทศของไทย คือ มี CD4 ต่ำกว่า 250 เซลล์/มม.^3^ สำหรับผู้ป่วยระยะที่ 2, 3 หรือ 4 ตามเกณฑ์ขององค์การอนามัยโลก หรือต่ำกว่า 200 ในกรณีที่ไม่แสดงอาการ ผู้ป่วยจะได้รับความรู้พื้นฐานเกี่ยวกับเอชไอวี โรคฉวยโอกาส การรักษาด้วยยาต้านไวรัส และความสำคัญของการทานยาตามกำหนด เพื่อที่พวกเขาจะสามารถทำการตัดสินใจได้อย่างมีข้อมูลว่า จะเริ่มการรักษาหรือไม่และเมื่อไหร่ ถ้าผู้ป่วยเข้ารับการรักษา การให้ยาต้านไวรัสก็จะดำเนินการโดยผู้ช่วยพยาบาลซึ่งเป็นผู้ต้องขังอาสาสมัครที่ช่วยเจ้าหน้าที่การแพทย์ในการจ่ายยาและตรวจวัดอุณหภูมิร่างกาย น้ำหนักและความดันโลหิต ยาสูตรแรกสำหรับผู้ป่วย 87 รายเป็นยาสูตรผสมตายตัว ประกอบด้วย stavudine, lamivudine และ nevirapine [9] ผู้ป่วยที่ติดเชื้อวัณโรคด้วยและรับยา rifampicin อยู่ จะได้รับยาต้านเป็น stavudine, lamivudine และ efavirenz

เรารับผู้ป่วยโดยจัดยาต้านไวรัสให้ 88 ราย ในจำนวนนี้ 12 ราย (15%) เคยรับยาต้านไวรัสมาก่อน แต่ขาดช่วงไปเนื่องจากการต้องขัง (ตาราง 1) ค่ามัธยฐานของช่วงระยะเวลาการติดตามผลคือ 18 เดือน และการเพิ่มระดับ CD4 นั้นได้ผลเทียบได้กับการรักษาภายใต้ตัวแปรแบบอื่น [9] ปัจจุบัน เราติดตามผู้ป่วย 63 ราย (72%) ที่ได้รับยาต้านไวรัสภายในเรือนจำ 18 ราย (20%) ได้รับการปล่อยตัว และ 5 ราย (6%) ถูกย้ายไปเรือนจำอื่น 2 ราย (2%) เสียชีวิต

การช่วยเหลือกันเองในหมู่ผู้ติดเชื้อฯ เป็นส่วนหนึ่งของยุทธศาสตร์การรักษาของประเทศไทย [10] และมีการใช้วิธีนี้ในเรือนจำทั้งสองแห่ง นอกจากการช่วยเหลือกันเองแล้ว ผู้ป่วยต่างมีเพื่อนคู่หูไว้คอยดูแลการทานยาตามกำหนด (เพื่อนคู่หูนี้ปกติแล้วเป็นผู้ติดเชื้อด้วยกันและอาจรับยาต้านไวรัสด้วยเช่นกัน)

กล่าวกันว่าเรือนจำเป็นสภาพแวดล้อมในอุดมคติสำหรับการปฏิบัติตามวิธีการรักษา[11] แต่จากประสบการณ์ของเรา ยังมีอุปสรรคที่สำคัญหลายประการที่ต้องฟันฝ่าที่ต้องอาศัยการประเมินอย่างต่อเนื่อง เป็นต้นว่า ความกลัวถูกตีตราบาปที่ทำให้ผู้ป่วยไม่ค่อยกล้าทานยาต่อหน้าคนอื่นๆ การไม่เชื่อใจเจ้าหน้าที่เรือนจำ รวมทั้งเจ้าหน้าที่โรงพยาบาล การถูกย้ายไปยังเรือนจำแห่งอื่น และการขาดการสนับสนุนทางสังคมเมื่อได้รับการปล่อยตัว

เจ้าหน้าที่ทางการแพทย์ในเรือนจำมีทัศนคติที่เป็นบวกมากต่อสิทธิในการรับยาต้านไวรัสของผู้ต้องขัง เจ้าหน้าที่มีความมุ่งมั่นในการทำงานฝ่าฟันอุปสรรคต่างๆ และนำผู้ป่วยส่งโรงพยาบาลอื่นในกรณีที่จำเป็นต้องใช้ความเชี่ยวชาญเฉพาะทาง การที่ไม่มีใครตั้งคำถามถึงการที่นักโทษประหารชีวิตสามรายได้รับยาต้านไวรัสเป็นข้อบ่งชี้ที่ชัดเจนถึงการมุ่งมั่นของเจ้าหน้าที่ที่ยึดถือว่าการรักษาเป็นสิทธิมนุษยชนขั้นพื้นฐาน

ทัศนคติที่เป็นบวกนี้เป็นสิ่งสำคัญต่อความสำเร็จในสภาพเงื่อนไขที่ความสัมพันธ์ระหว่างผู้ต้องขังและเจ้าหน้าที่เรือนจำมีความเป็นปฏิปักษ์ต่อกันนับแต่ไรมา ในอดีตนั้นผู้ต้องขังไม่เชื่อใจเจ้าหน้าที่และไม่เต็มใจที่จะเข้ารับการดูแลรักษา ผู้อำนวยการการแพทย์ที่บางขวางบอกว่า ต้องใช้เวลาสองปีถึงจะทำให้ผู้ป่วยเกิดความเชื่อใจแล้วมาขอรับการรักษา

**การโอนย้ายและการปล่อยตัวผู้ป่วย**

เรือนจำที่กรุงเทพฯ มีผู้ต้องขังแออัดยัดเยียด จึงมักมีการย้ายผู้ต้องขังไปยังเรือนจำต่างจังหวัดที่แออัดน้อยกว่า การย้ายดังกล่าวสร้างปัญหาต่อความต่อเนื่องของการรักษาเนื่องจากเรือนจำต่างจังหวัดส่วนใหญ่มีเจ้าหน้าที่การแพทย์น้อยและไม่มีการรักษาเอชไอวี ถึงแม้จะได้มีการพยายามสื่อสารสร้างความตระหนักถึงความจำเป็นในการดูแลรักษาผู้ติดเชื้อเอชไอวี/เอดส์เป็นการเฉพาะภายในระบบเรือนจำแล้วก็ตาม เราก็ยังไม่ทราบได้ว่าผู้ป่วย 5 รายของเราที่ถูกย้ายไปเรือนจำอื่นนั้นได้รับการดูแลรักษาอย่างไร

ความต่อเนื่องของการรักษาเมื่อพ้นโทษอาจมีปัญหาจากการใช้สารเสพติด การไร้บ้าน การว่างงาน ความยากจน และอุปสรรคในการเข้าถึงโครงการประกันสุขภาพ โดยเฉพาะสำหรับอดีตผู้ต้องขังที่ไม่มีบัตรประจำตัวประชาชน (ล้อมกรอบ 3) ก่อนที่จะถูกปล่อยตัวจากเรือนจำ ผู้ป่วยได้พบกับนักสังคมสงเคราะห์ของ MSF เพื่อวางแผนทำการรักษาอย่างต่อเนื่อง ในเรือนจำทั้งสองแห่ง MSF จัดหายาให้เป็นเวลา 3 เดือน ซึ่งเป็นระยะเวลาโดยเฉลี่ยสำหรับคนที่มีบัตรประจำตัวประชาชนที่เข้าร่วมโครงการรักษาด้วยยาต้านไวรัสของรัฐบาล จากผู้ต้องขังจำนวน 18 รายที่ได้รับการปล่อยตัวตั้งแต่เริ่มโครงการรักษา มี 7 รายที่ได้รับการรักษาผ่านระบบสาธารณสุข ขณะที่ 6 รายรับการรักษาจาก MSF เพราะว่าไม่มีบัตรประชาชน ส่วนอีก 5 รายขาดการติดต่อไป แม้ว่าจะได้มีการพยายามติดต่อแล้วก็ตาม

**ข้อพิจารณาเชิงจริยธรรมที่เกี่ยวข้องกับบทความนี้**

ในการเข้าไปข้องเกี่ยวใดๆ ก็ตามกับกลุ่มประชากรที่ถูกจำกัดเสรีภาพ ย่อมจะต้องเกิดประเด็นคำถามเชิงจริยธรรมขึ้นมาอย่างเลี่ยงไม่พ้น ในบทความนี้ เราได้บรรยายประสบการณ์ในการดำเนินการยุทธศาสตร์ป้องกันเอชไอวีที่ได้รับการพิสูจน์แล้ว และให้การรักษาระดับมาตรฐานแก่กลุ่มคนที่เข้าถึงได้ยาก โดยต้องคิดหาวิธีการขึ้นมาใหม่ ประเด็นท้าทายเชิงจริยธรรมหลักๆ ที่เราได้เผชิญร่วมกับผู้ร่วมงานนั้นคือ การเก็บข้อมูลผู้ป่วยเป็นความลับและการยินยอมอย่างมีข้อมูลประกอบการตัดสินใจ เราได้พยายามจัดการกับประเด็นท้าทายเหล่านี้อย่างดีที่สุดเท่าที่จะทำได้

ในช่วงการพิจารณาทบทวนเนื้อหาบทความในคณะผู้เขียนด้วยกัน มีการหยิบยกประเด็นทางจริยธรรมขึ้นมาพูดคุยกันหลายประเด็น โดยเฉพาะประเด็นการปกปิดตัวตนของผู้ป่วยและการขอความเห็นชอบสำหรับการตีพิมพ์ เรื่องนี้ได้รับการนำไปหารือกับคณะกรรมการที่ปรึกษาด้านจริยธรรมทางการพิมพ์ของ *PLoS Medicine* ซึ่งก็ยังไม่มีความเห็นพ้องต้องกัน[12] เราหารือเรื่องนี้กับคณะกรรมการอิสระ Institutional Review Board ซึ่งลงความเห็นว่าการตีพิมพ์บทความนี้มีความเสี่ยงน้อยมากที่จะก่อให้เกิดผลกระทบต่อบุคคลหรือชุมชนนั้น คณะกรรมการยังรู้สึกด้วยว่าด้วยการที่เป็นบทความบรรยายที่ไม่ได้มีการพิสูจน์สมมติฐานการวิจัยใดๆ และไม่ได้มีการใช้สถิติเชิงวิเคราะห์ ดังนั้นจึงไม่มีความจำเป็นที่จะต้องให้คณะกรรมการจริยธรรมพิจารณาและเห็นชอบอย่างเป็นทางการ

**ข้อถกเถียง**

ในประเทศกำลังพัฒนามีตัวอย่างโครงการรักษาผู้ติดเชื้อเอชไอวี/เอดส์อยู่น้อยมาก ผลที่ได้จากโครงการนำร่องต่างๆ ล้วนสนับสนุนความมีประสิทธิผลของการรักษาในเรือนจำ [13-15] แต่ก็ยังมีรายงานอยู่เสมอถึงการขาดแคลนยาต้านไวรัสหรือไม่มียาเลย [16-18] การขาดแคลนนี้ได้กลายเป็นข้อกังวลมากขึ้นเรื่อยๆ สำหรับกิจกรรมการรักษาต่างๆ อย่างเช่น ในแอฟริกาใต้ เมื่อเร็วๆ นี้กลุ่มประชาสังคมได้ยื่นฟ้องรัฐบาลต่อศาลเพื่อต่อสู้เพื่อสิทธิในการรักษาของผู้ต้องขัง[19]

จากประสบการณ์ของเรา การบรรลุผลลัพธ์ที่น่าพอใจนั้นสามารถกระทำได้ภายใต้สภาพเงื่อนไขของสถานที่คุมขังที่ขาดแคลนทรัพยากร ในระยะเริ่มแรก MSF ทำหน้าที่จัดหาการรักษาสำหรับโรคฉวยโอกาส ยาต้านไวรัสและการสนับสนุนด้านเทคนิค ประเทศไทยไม่ใช่ประเทศ “ด้อยพัฒนา” ทว่าวิธีการที่เลือกใช้นั้นเป็นไปตามแนวทางขององค์การอนามัยโลกสำหรับการรักษาพยาบาลในสภาพเงื่อนไขที่มีทรัพยากรอัตคัด และสามารถดำเนินการโดยรัฐบาลใดก็ได้ในการให้การรักษาในหมู่ประชากรทั่วไป ถึงที่สุดแล้ว อุปสรรคที่ขัดขวางการให้การรักษาในเรือนจำขณะที่ข้างนอกมีการรักษานั้นไม่ใช่เป็นปัญหาทางเทคนิค หรือทางการเงิน หากแต่เป็นทางการเมือง

ประสิทธิผลของโครงการใดๆ ในเรือนจำนั้นขึ้นอยู่กับทัศนคติของเจ้าหน้าที่เป็นสำคัญ เราได้พบว่าทัศนคติของเจ้าหน้าที่ด้านการแพทย์ของเรือนจำต่อสิทธิของผู้ต้องขังนั้นเป็นไปในทางบวกมาก แต่ก็ไม่ใช่เจ้าหน้าที่ทุกคน การจัดหาให้เกิดการรักษาเป็นก้าวสำคัญก้าวแรกในการสร้างความไว้เนื้อเชื่อใจที่จะทำให้เราสามารถเข้าไปแตะประเด็นที่ละเอียดอ่อนกว่าในเรื่องการป้องกันได้

เราเชื่อว่าโครงการนี้จะส่งผลสะเทือนที่ยั่งยืน ในส่วนของการป้องกันนั้นรวมถึงยุทธศาสตร์การเปลี่ยนทัศนคติและพฤติกรรมของทั้งผู้คุมและผู้ต้องขัง และทางกรมราชทัณฑ์ได้ขอให้เราพัฒนาหลักสูตรอบรมสำหรับเรือนจำอื่นๆ ด้วย ในส่วนของการรักษานั้น หน้าที่การจัดหายาได้ส่งมอบไปอยู่ในความดูแลของรัฐบาล การฝึกอบรมและการเป็นพี่เลี้ยงที่ผ่านมาได้ทำให้เจ้าหน้าที่การแพทย์ของเรือนจำมีทักษะในการจัดการกับปัญหาส่วนใหญ่ได้แล้ว และความยั่งยืนยังเกิดจากการที่งบประมาณส่วนหนึ่งสำหรับการดูแลรักษาผู้ต้องขัง รวมถึงยาต้านไวรัสด้วย ขณะนี้อยู่ภายใต้โครงการประกันสุขภาพ แต่อย่างไรก็ตาม ชนกลุ่มน้อยและชาวต่างชาติยังไม่ได้รับการคุ้มครอง และประเด็นการเข้าถึงการรักษาของพวกเขาเป็นข้อกังวลที่เร่งด่วน

ผู้ต้องขังส่วนใหญ่มาจากกลุ่มคนชายขอบ อย่างเช่น ผู้ติดยาเสพติด ผู้ให้บริการทางเพศ หรือแรงงานต่างชาติที่ไม่ได้ลงทะเบียน ซึ่งมีความเสี่ยงสูงต่อเอชไอวีและมีข้อจำกัดในการเข้าถึงการรักษาโดยทั่วไป และเรื่องนี้แสดงให้เห็นจากผู้ติดเชื้อเอชไอวีจำนวนมากที่พบในเรือนจำ การทำงานในเรือนจำเปิดโอกาสให้ได้ทำงานกับกลุ่มคนเฉพาะบางกลุ่มที่ปกติแล้วจะไม่เข้ารับ(หรือไม่ได้รับ)การรักษา การจะรับมือปัญหาให้ได้อย่างมีประสิทธิผลจึงจะต้องรวมถึงการจัดการอุปสรรคของการรักษาภายนอกเรือนจำด้วยเพื่อให้การรักษามีความต่อเนื่องสำหรับผู้ที่ได้รับการปล่อยตัว

**กิตติกรรมประกาศ**

เราขอขอบคุณคุณนิภา งามไตรไร (กรมราชทัณฑ์), นพ.มานพ ศรีสุพรรณถาวร และ คุณบุญยัง ฉายาทัพ (เรือนจำกลางบางขวาง) และ คุณสุชาดา โพธิ์กิ่ง คุณนิตยา อินคา และคุณคุณากร วิริยะรัมภะ (เรือนจำพิเศษมีนบุรี) สำหรับการสนับสนุนและข้อเสนอแนะสำหรับบทความนี้ โดยเฉพาะอย่างยิ่งขอขอบคุณผู้ต้องขังที่ได้ให้ข้อมูลอันเป็นประโยชน์ ซึ่งจำเป็นต้องขอสงวนนาม นอกจากนี้ก็ขอขอบคุณ Philippe Cavailler สำหรับการให้คำแนะนำเกี่ยวกับการเก็บข้อมูลและ Dr France Roblain ท้ายที่สุดนี้ เราขอขอบคุณทุกคนที่ได้อุทิศเวลาในการวิจารณ์เสนอแนะปรับปรุงบทความร่างแรกของเรา

**ผู้เขียน** *เดวิด วิลสัน* และ *อาร์ลีน ชัว* ออกแบบงานวิจัย *นาธาน ฟอร์ด วีระพันธ์ งามมี* และ *อาร์ลีน ชัว* ร่วมเขียนบทความ *อาร์ลีน ชัว* และ *โม โจ โจ้* วิเคราะห์ข้อมูล *เดวิด วิลสัน* และ *อาร์ลีน ชัว* พิจารณารับคนไข้เข้าโครงการ *เดวิด วิลสัน วีระพันธ์ งามมี อาร์ลีน ชัว* และ *โม โจ โจ้* รวบรวมข้อมูล *เดวิด วิลสัน* และ *อาร์ลีน ชัว* ให้การดูแลรักษาคนไข้ จ่ายยา และลงบันทึก รวมทั้งเป็นที่ปรึกษาแก่แพทย์ในโครงการ *เดวิด วิลสัน* พิจารณาและวิเคราะห์ข้อมูลทุก 3 เดือน *เดวิด วิลสัน* เป็นผู้อำนวยการด้านการแพทย์ขององค์การหมอไร้พรมแดน-เบลเยี่ยม (ประเทศไทย) และมีส่วนในการออกแบบกรอบการศึกษา และวิเคราะห์ข้อมูล ผู้เขียนอีกสองท่านร่างรายงานฉบับแรก (รายงานภายในองค์กร) และ *เดวิด วิลสัน* ปรึกษาหารือและปรับปรุงร่าง จนได้บทความวิชาการร่างสุดท้ายเพื่อออกเผยแพร่ *วีระพันธ์ งามมี* เป็นผู้ประสานงานโครงการในพื้นที่ที่อยู่ในบทความ และ มีส่วนสำคัญในการออกแบบการวิจัย ประเมินผล และ ติดตาม *อาร์ลีน ชัว* เป็นแพทย์ที่รักษาผู้ป่วยในโครงการตั้งแต่เดือนมิถุนายน 2546 ถึง ถึงเดือนธันวาคม 2548 *โม โจ โจ้* วิเคราะห์ข้อมูลด้วยโปรแกรม “FUCHIA” ซึ่งทาง MSF ใช้ในการติดตามและประเมินผลในโครงการการให้ยาต้านไวรัส

, , , ,

# ตาราง 1: ข้อมูลและผลการรักษาของผู้ป่วยเอชไอวีที่ได้รับยาต้านไวรัสในเรือนจำทั้งสองแห่ง

| ข้อมูลผู้ป่วย | **ผู้ป่วยที่รับยาต้านไวรัส (จำนวน 88 ราย)** |
| --- | --- |
| ชาย | 72 (82%) |
| หญิง | 16 (18%) |
| อายุเฉลี่ย (ต่ำสุด – สูงสุด) | 34 (21-49) |
| ระยะเวลาการติดตามมัธยฐาน (เดือน) (IQR) | 18 (9-23) |
| ระยะของอาการตามเกณฑ์องค์การอนามัยโลก เมื่อเริ่มรับยาต้านไวรัส |  |
| - ระยะที่ 1 | 7 (8%) |
| - ระยะที่ **2** | 10 (11%) |
| - ระยะที่ 3 | 41 (47%) |
| - **ระยะที่ 4** | 30 (34%) |
| CD4 มัธยฐาน เมื่อเริ่มรับยาต้านไวรัส (IQR) | 119 (23-205) |
| ผล |  |
| CD4 มัธยฐานเพิ่มขึ้น (จำนวน 14 ราย : ไม่รวมกรณีที่ไม่มีผลการตรวจ |  |
| - หลังรับยาต้านไวรัส 6 เดือน | 73.5 (ความคลาดเคลื่อนมาตรฐาน 14.1) |
| - หลังรับยาต้านไวรัส 12 เดือน | 134 (ความคลาดเคลื่อนมาตรฐาน 24.6) |
| - หลังรับยาต้านไวรัส 18 เดือน | 161 (ความคลาดเคลื่อนมาตรฐาน 35.9) |
| เปลี่ยนยาต้านไวรัส (จำนวน 11 ราย) |  |
| ดื้อยา stavudine (เปลี่ยนเป็น zidovudine) | 4 |
| ดื้อยา nevirapine (เปลี่ยนเป็น efavirenz) | 6 |
| เปลี่ยนเป็นสูตรสำรอง (ล้มเหลวยืนยันด้วย Viral Load) | 1 |

IQR=Inter quartile range

**ล้อมกรอบ 1**: **เรือนจำกลางบางขวางและเรือนจำพิเศษมีนบุรี**

| เรือนจำพิเศษมีนบุรีเป็นเรือนจำระดับกลาง รองรับผู้ต้องขังได้ 2,000 คน (ชาย 1,700 หญิง 300) ระยะเวลาการต้องโทษอยู่ระหว่าง 2-7 ปี เจ้าหน้าที่การแพทย์ประกอบด้วยแพทย์ที่ทำงานครึ่งเวลาหนึ่งคน และพยาบาลเต็มเวลาสามคน ผู้ต้องขังที่ต้องได้รับการรักษาพยาบาลถูกส่งไปที่โรงพยาบาลกลาง ซึ่งเป็นโรงพยาบาลขนาด 300 เตียงในเรือนจำกลางคลองเปรมที่กรุงเทพฯ MSF เริ่มทำงานในเรือนจำพิเศษมีนบุรีในเดือนมิถุนายน พ.ศ. 2546  เรือนจำกลางบางขวางเป็นเรือนจำสำหรับผู้ต้องขังคดีอุกฉกรรจ์ รองรับผู้ต้องขังได้ 3,500 คน (ชายล้วน) แต่ในเดือนมีนาคม 2549 ปรากฏมีผู้ต้องขังอยู่ถึง 4,922 คน รวมนักโทษประหาร 870 คน โทษจองจำต่ำสุดคือ 25 ปี ภายในมีโรงพยาบาลขนาด 40 เตียงและคลินิกรับคนไข้นอก มีแพทย์เต็มเวลาหนึ่งคน (ผู้อำนวยการฝ่ายการแพทย์ของเรือนจำ) แพทย์ไม่เต็มเวลาหนึ่งคน พยาบาล 6 คน พยาบาลเทคนิค 6 คน เภสัชกร 2 คน เจ้าหน้าที่ห้องแล็บ 1 คนและเจ้าหน้าที่เอ็กซ์เรย์ 2 คน MSF เริ่มทำงานที่บางขวางในเดือนธันวาคม 2547  เรือนจำทั้งสองแห่งมีจำนวนผู้ต้องขังล้นเกินความสามารถในการรองรับ ผู้ต้องขังอยู่ร่วมกันในห้องขังขนาดใหญ่ ที่มีนบุรีห้องขังหนึ่งมีผู้ต้องขัง 400 คนสำหรับผู้ชาย 300 คนสำหรับผู้หญิง ที่บางขวาง มีผู้ต้องขัง 20 – 40 คนต่อห้อง ยกเว้นแดนประหารซึ่งมีห้องละ 100 คน มีผู้ต้องขังส่วนหนึ่งจำนวนไม่มากนักถูกขังเดี่ยว  ในเรือนจำทั้งสองแห่ง ผู้ต้องขังจะต้องอยู่ภายในห้องขังตั้งแต่เวลา 16.00 น. ถึง 7.00 น. ซึ่งระหว่างนี้เจ้าหน้าที่การแพทย์ไม่สามารถเข้าไปในห้องขังได้ ที่บางขวาง มีผู้ต้องขังอาสาสมัครทำหน้าที่ทำการ “ปฐมพยาบาล” ในช่วงเวลาดังกล่าว |
| --- |

ล้อมกรอบ 2: เพศสัมพันธ์ภายในเรือนจำ

| เพศสัมพันธ์ในเรือนจำชายเป็นอย่างไร?   - **เพศสัมพันธ์ในเรือนจำมักจะเป็นเรื่องของอำนาจ** - **เพศสัมพันธ์ที่*ยินยอม*ระหว่างชายมีทั้ง เพศสัมพันธ์คู่ขา การซื้อขายบริการทางเพศ และการมีเพศสัมพันธ์เพื่อแลกกับการคุ้มครอง** - **เพศสัมพันธ์ที่*ไม่ยินยอม*มีทั้ง การข่มขืน บางครั้งใช้เป็นกลยุทธ์การข่มขู่ และการบังคับใช้หนี้ ผู้ต้องขังรายใหม่จะตกเป็นเป้าพิเศษ เนื่องจากต้องมีการรับน้องเพื่อแสดงความสัมพันธ์เชิงอำนาจภายในเรือนจำ**   ทำไมทัศนคติของเจ้าหน้าที่เรือนจำจึงอาจเป็นอุปสรรคขัดขวางการมีเพศสัมพันธ์ที่ปลอดภัยมากขึ้น?   - **เจ้าหน้าที่บางคนพยายามห้ามกิจกรรมทางเพศทุกชนิดเพื่อป้องกันการบังคับมีเพศสัมพันธ์ คนอื่นๆ ก็เชื่อว่าการห้ามการมีเพศสัมพันธ์เป็นมาตรการป้องกันที่ได้ผลมากที่สุด แม้ว่าจะไม่เป็นจริงในทางปฏิบัติ**   ทำไมผู้ต้องขังจึงไม่ได้ใช้ถุงยางอนามัย?   - **ถุงยางอนามัยไม่ใช่สิ่งต้องห้ามในเรือนจำ แต่ในทางปฏิบัติ ผู้คุมมักไม่ยอมรับการแจกจ่ายถุงยาง** - **ผู้ต้องขังบางคนไม่ทราบถึงความเสี่ยงของการแพร่เชื้อเอชไอวีจากการร่วมเพศทางทวารหนัก คนอื่นๆ ก็ทราบ แต่ไม่สามารถหาถุงยางหรือต่อรองให้ใช้ถุงยางในสถานการณ์นั้นได้** - **การมีถุงยางหรือให้คนอื่นรู้ว่าตนมีเพศสัมพันธ์ อาจสร้างความอับอายต่อเพื่อนผู้ต้องขังหรือผู้คุมไม่พอใจได้ ผู้ต้องขังจึงอาจยอมที่จะเสี่ยงติดเชื้อดีกว่าพยายามหาถุงยาง** - **ผู้ต้องขังอาจเชื่อว่าการมีเพศสัมพันธ์กับผู้ชายด้วยกันเป็นการผิดธรรมชาติ ทำให้พวกเขาต้องเก็บงำ และแอบมีเพศสัมพันธ์โดยไม่มีการป้องกัน** - **คนที่ยืนยันให้ใช้ถุงยางอาจจะถูกเข้าใจว่ามีเชื้อเอชไอวีหรือเชื้ออื่นๆ ได้** |
| --- |

ล้อมกรอบ 3: ความต่อเนื่องของการรักษาหลังได้รับการปล่อยตัว: กรณีศึกษา

ว.บ.เป็นชายไทยอายุ 33 ปีที่ทำบัตรประชาชนหายไปเมื่อหลายปีก่อน เขาพบว่าติดเชื้อเอชไอวี ขณะอยู่ในเรือนจำพิเศษมีนบุรี และเริ่มรับการรักษาด้วยยาต้านไวรัสในเดือนพฤษภาคม 2547 เขาติดต่อกับ MSF หลายครั้งเพื่อให้มั่นใจว่าเขาจะยังได้รับการรักษาอย่างต่อเนื่องหลังพ้นโทษ เขาได้รับการปล่อยตัวเมื่อเดือนตุลาคม 2548 พักอยู่กับเพื่อนที่เขาไม่ได้บอกให้ทราบถึงการติดเชื้อของเขา เขาทำงานเป็นช่างเชื่อม MSF จัดหายาให้เขาและจัดการการตรวจสอบเฝ้าระวัง เขาต้องหาเอกสารเพื่อทำบัตรประชาชน ประกอบด้วย สูติบัตร มรณะบัตรของบิดา ใบรับรองการปล่อยตัวจากเรือนจำ การรับรองการพักอาศัย ซึ่งต้องให้เจ้าของบ้านเพิ่มชื่อเขาเข้าไปในทะเบียนบ้าน และใบรับรองความประพฤติสองฉบับที่ลงนามโดยข้าราชการ MSF ช่วยเขายื่นเรื่องตามกระบวนการ และเขาได้รับบัตรประชาชนในเดือนกุมภาพันธ์ 2550 เป็นเวลา 16 เดือนนับจากพ้นโทษ ทำให้เขาสามารถเข้าโครงการประกันสุขภาพของรัฐได้ หลังจากนั้นอีกสามเดือน เขาก็ได้รับการรักษาผ่านโครงการของรัฐบาล

C.S. เป็นชายอายุ 30 ปี พบว่าติดเชื้อเอชไอวีในปี 2545 ในเรือนจำมีนบุรี เขาไม่ได้เข้ารับการรักษาขณะอยู่ในเรือนจำ แต่ขอให้ MSF มาเยี่ยมเขาเมื่อได้รับการปล่อยตัวในเดือนพฤษภาคม 2547 หลังจากนั้น ก็พบว่าเขาเป็นโรค miliary tuberculosis แล้วก็เริ่มรับการรักษาด้วยยาต้านไวรัสในเดือนพฤษภาคม 2548 หลังจาก MSF ช่วยให้เขาเข้าโครงการรักษาด้วยยาต้านไวรัสของรัฐบาล เขาเคยติดยาเสพติด และกลับไปติดอีกครั้งในเดือนมิถุนายน 2548 อย่างไรก็ตาม เขายังทานยาตามกำหนด

**References:**

1. Jürgens R. From Evidence to Action on HIV/AIDS in Prisons: A Report From the XVI International AIDS Conference. Infectious Diseases in Corrections Report, September 2006. Main Article 1. <http://www.idcronline.org/>

2. UNAIDS/WHO. AIDS Epidemic Update. Geneva, December 2005

3. External review of the health sector response to HIV/AIDS in Thailand. Ministry of Health/World Health Organisation. 7-19 August 2005.

4. World Prison Brief. International Centre For Prison Studies, Kings College, London. [www.prisonstudies.org](http://www.prisonstudies.org/) (accessed 02 March 2006)

5. Thailand's Response to HIV/AIDS: Progress and Challenges. United Nations Development Programme, New York, 2004.

6. Thaisri H, Lerwitworapong J, Vongsheree S, Sawanpanyalert P, Chadbanchachai C, Rojanawiwat A, Kongpromsook W, Paungtubtim W, Sri-ngam P, Jaisue R. HIV infection and risk factors among Bangkok prisoners, Thailand: a prospective cohort study. *BMC Infectious Diseases* 2003, 3:25.

7. Beyrer C, Jittiwutikarn J, Teokul W, Razak M, Suriyanon V, Srirak N, Vongchuk T, Tovanabutra S, Sripaipan T, Celentano D. Drug use, increasing incarceration rates and prison-associated HIV risks in Thailand. *AIDS and Behaviour* 2003. 7;2:153-161.

8. www.correct.gov.th/statis.htm

9. Generic fixed-dose combination antiretroviral treatment in resource-poor settings: multicentric observational cohort. Alexandra Calmy, Lorextu Pinoges, Elizabeth Szumilin, Rony Zachariah, Nathan Ford and Laurent Ferradini. *AIDS* 2006;20:1163–1169

10. Kumphitak A, Kasi-Sedapan S, Wilson D, Ford N, Adpoon P, Kaetkaew S, Praemchaiporn J, Sae-Lim A, Tapa S, Teemanka S, Tienudom N, Upakaew K. Perspectives and Practice in Antiretroviral Treatment: Involvement of people living with HIV/AIDS in Treatment Preparedness. MSF/TNP+/AAF. World Health Organization, Geneva, 2004. Available at: <http://www.who.int/hiv/pub/prev_care/en/thailand.pdf>

11. Dixon PS, Flannigan TP, DeBuono BA, Laurie JJ, Hoy J, Stein M, et al. Infection with the human immunodeficiency virus in prisoners: meeting the health care challenge. *Am J Med* 1993;95:629-635.

12. The PloS Medicine Editors. PloS Medicine's Advisory Group on Publication Ethics. *PloS Medicine* 2007;4:0201-0202

13. Simooya O, Sanjobo N. Challenges and opportunities for scaling up HIV/AIDS care in prisons: A case study from Zambia. XVI International AIDS Conference, Toronto, 2006. Abstract no. TUAX0102.

14. Akpan RC. HIV/AIDS intervention program in the prisons communities in Nigeria. Presented at "HIV/AIDS in Prison: A Comprehensive Response", satellite meeting at the XVI International AIDS Conference, 14 August 2006.

15. Chepkonga MC. Kenya Prisons Service HIV/AIDS Program. Presented at "HIV/AIDS in Prison: A Comprehensive Response", satellite meeting at the XVI International AIDS Conference. 2006.

16. Rwanda: Guarding against HIV in prisons. Plus News, 24 August 2006.

17. Kenya: HIV-positive prisoners often locked out of services. Plus News, 14 June 2006.

18. Cameroon: Tuberculosis and AIDS soaring in prisons. Plus News, 23 February 2006.

19. SA could face constitutional crisis: judge. SAPA, August 26, 2006.
